# Are all health gains equally important? An exploration of acceptable health as a reference point in health care priority setting

**DOI:** 10.1186/s12955-015-0277-6

**Published:** 2015-06-10

**Authors:** S. Wouters, N.J.A. van Exel, K.I.M. Rohde, W.B.F. Brouwer

**Affiliations:** Institute of Health Policy & Management, Erasmus University Rotterdam, 3000 DR Rotterdam, The Netherlands; Erasmus School of Economics, Erasmus University Rotterdam, 3000 DR Rotterdam, The Netherlands

**Keywords:** Acceptable health, Reference point, Resource allocation, Ageing, 'Normal' functioning

## Abstract

**Background:**

Accumulating evidence suggests that members of society prefer some QALY gains over others. In this paper, we explore the notion of acceptable health as a reference point in assessing the value of health gains. The value of health benefits may be assessed in terms of their position relative to this reference level, benefits above the level of acceptable health being valued differently from benefits below this level. In this paper we focus on assessing the level of acceptable health at different ages and associations with background variables.

**Methods:**

We recruited a sample of the adult population from the Netherlands (n = 1067) to investigate which level of health problems they consider to be acceptable for people aged 40 to 90, using 10-year intervals. We constructed acceptable health curves and associated acceptable health with background characteristics using linear regressions.

**Results:**

The results of this study indicate that the level of health problems considered acceptable increases with age. This level was associated with respondents’ age, age of death of next of kin, health and health behaviour.

**Conclusions:**

Our results suggest that people are capable of indicating acceptable levels of health at different ages, implying that a reference point of acceptable health may exist. While more investigation into the measurement of acceptable health remains necessary, future studies may also focus on how health gains may be valued relative to this reference level. Gains below the reference point may receive higher weight than those above this level since the former improve unacceptable health states while the latter improve acceptable health states.

## Background

Scarcity of health care resources makes priority setting inevitable. An increasingly used tool to inform such decision-making is cost-utility analysis, which assesses the incremental costs and health benefits of a new intervention relative to some relevant alternative (like an old intervention, doing nothing or care as usual). The results of a cost-utility analysis are typically summarized in an incremental cost-utility ratio (ICUR), expressing the incremental costs per unit of health gain [1]. Costs are expressed in monetary terms while health gains are generally expressed in terms of quality-adjusted life years (QALYs), i.e. the amount of life time gained by the intervention corrected for the quality of life (QoL) during that time, with a QoL score of 1 representing perfect health and a QoL score of 0 representing the state of being dead [2]. An intervention may be considered to offer value for money and hence be considered for reimbursement when its costs per QALY are lower than some relevant threshold [1]. The nature and height of this threshold are a matter of ongoing discussion (e.g. [3, 4]).

In calculating the cost-utility of interventions in health care, each QALY is usually weighted equally, regardless of whom it accrues to or under which circumstances it is gained. This means that in calculating QALY gains, it does not matter whether, for example, a QALY is gained in the context of a severe or mild illness or whether the beneficiary is 10 or 80 years old. However, accumulating evidence (from the literature and policy practice) suggests that members of society do prefer some QALY gains over others. Disease and patient characteristics such as the severity of the treated illness and the age of the beneficiaries have been found to be important in the valuation of health gains [5–7]. This means that priority setting based on QALY maximization is unlikely to reflect societal preferences for the just distribution of health and health care, and that weighting QALYs according to particular equity principles may improve the societal support for health care decisions.

Two prominent equity principles that may justify and guide the process of empirically deriving such equity weights are the fair innings principle [8] and the severity of illness argument [9–11]. Fair inning aims to promote equality in lifetime health, assigning higher priority to people who have not had their ‘fair share’ of lifetime health than to people who have, and therefore live on ‘borrowed time’. The severity argument aims to promote equality in people’s prospective health, therefore assigning higher priority to those people whose health status or expectations are worse [7].

Notwithstanding the increasing focus on equity considerations, an underexplored element for QALY weighting relates to the notion of acceptability of imperfect health states. Brouwer et al. [12] suggested and empirically tested the notion of acceptable health states in relation to age. They argued that some health problems may be considered a normal part of ageing, hence making them acceptable beyond a certain age. For example, we may be inclined to view impairment in mobility (e.g. being unable to walk long distances) to be unacceptable for the average 20-year old person, while we may consider this to be quite acceptable for the average 90-year old person. In their study, Brouwer et al. [12] indeed found that people considered an increasing number and level of health problems acceptable as age progresses. Likewise, Stolk et al. [13] argue that one of the reasons for resistance against the funding of Viagra in The Netherlands was that it was considered as ‘unnecessary luxury care’, because erectile dysfunction was seen as a normal and acceptable consequence of ageing.

Such reasoning implies that some imperfect but still acceptable health state is used as a reference point [14] in assessing the necessity of treatment or the value of health gains. Taking such a reference point implies that health gains that accrue to people whose health is below the reference point, and thus given their age are in ‘unacceptable health states’, will carry more weight than equally sized gains accruing to people who are already above this reference point. This differs from the conventional way of dealing with QALY gains, in which all deviations from perfect health are seen as losses and are weighted equally.

The notion of acceptability carries elements of both the severity of illness and the fair innings arguments. The severity of illness argument suggests that health gains lower on the QoL scale carry more weight than those high on the scale. Hence, treatments for more severe diseases will get higher priority than those aimed at milder diseases. The acceptability argument refines this argument by stating that gains above a specific reference point of acceptability receive less priority than those below this reference point. Health gains may accordingly be assigned different social values. In line with the fair innings argument, the reference levels of acceptable health may be age dependent. This means that acceptable deviations from perfect health may be larger for older than for younger people. A difference with the fair innings argument is that this age-dependent reference point may be independent from health achievements in the past and solely focus on peoples’ health at a specific point in time.

In allocation decisions the notion of acceptable health states can lead to a change in the value that health gains receive. A first step in operationalising the notion of acceptable health as a reference point is to investigate whether people indeed consider some imperfect health states to be acceptable and whether this depends on age. This paper focuses on that question by investigating the acceptability of imperfect health states for people aged 40 to 90 in a representative sample of the population between the ages of 18 and 65 from the Netherlands. In addition, respondents were asked what they considered to be an acceptable age of death, which allowed us to estimate the acceptable amount of lifetime QALYs after the age of 40 years. This could be seen as a specific operationalisation of ‘fair innings’ for lifetime health achievement. Furthermore, we investigated which socio-demographic characteristics are associated with the elicited acceptability levels, as well as respondents’ own health and health behaviour and the age of death of their next of kin.

## Methods

In order to investigate whether certain imperfect health states are considered acceptable, a professional sampling agency recruited 1067 respondents between the ages of 18 and 65. Invitations to complete a web-based questionnaire were sent out to members of the recruitment agency’s panel. Members of this panel had previously signed up to participate in surveys and experiments, and by accepting the invitation to participate in this survey provided consent to the use of their response for the purposes of this study. Strategic sampling was used to obtain a sample representative for the Dutch general public between the ages of 18 and 65 in terms of age, gender and education level. The data collection was part of a larger research project exploring people’s expectations of length and quality of life.

In the questionnaire, respondents were asked to indicate which level of health problems they considered to be acceptable from the ages 40, 50, 60, 70, 80 and 90 years onwards. They were also asked what they considered to be an acceptable age of death. In addition, the questionnaire included questions regarding respondents’ socio-demographic characteristics, their current health status, their health lifestyle in terms of exercise, nutrition, smoking and alcohol intake, and the age of death of their next of kin (i.e., their parents, grandparents, aunts and uncles, and other family members).

Similar to Brouwer et al. [12], the health problems that respondents evaluated in terms of their acceptability were described using the EQ-5D-3L descriptive system [15]. The EQ-5D-3L describes health states using five domains (i.e. mobility, self-care, usual activities, pain/discomforts and anxiety/depression) and distinguishes three levels within each domain (i.e. no problems, some problems and severe problems). Different combinations of these three levels in the five domains allow the EQ-5D-3L to describe 243 distinct health states. These are often labeled using a 5 digit code like 11233, which refers to a health state with no problems with mobility and self-care (i.e. level 1 on domains 1 and 2), some problems with usual activities (i.e. level 2 on domain 3) and severe pain/discomfort and anxiety/depression (i.e. level 3 on domains 4 and 5). Health-related quality of life scores can be computed for each health state using validated Dutch EQ-5D-3L utility tariffs [16, 17]. The utility scores take values between 1 for perfect health and 0 for dead, while health states considered worse than dead receive a negative utility score.

In this study, respondents were asked to indicate in each of the EQ-5D-3L domains from which age onwards they considered the levels ‘some problems’ and ‘severe problems’ acceptable (see Appendix). Acceptable levels of health were computed for the ages 40 to 90 by combining the answers that respondents gave in each separate domain. These scores were then used to construct an acceptable health curve (AHC_AGGREGATE_), defined by the sample average acceptable quality of life score at each age.

Combining the separate domain-specific responses in this way had the advantage that it allowed us to analyse the acceptability of each of the 243 health states described by the EQ-5D-3L, but had the disadvantage that it may have overestimated the acceptability of health states. Since respondents evaluated health problems in each domain in isolation, they may not have taken simultaneous health problems in other domains into account. For example, some problems with mobility or with self-care may be considered acceptable at the age of 60 when evaluated in isolation, but health state 22111 may nonetheless be considered unacceptable at that age. Therefore, we constructed a more conservative acceptable health curve (AHC_WORST_), based only on the worst health problem in any 1 of the 5 domains considered to be acceptable at the different ages. For instance, in case health state 32211 was acceptable at the age of 70 based on a respondent’s separate answers per health domain, AHC_AGGREGATE_ was calculated based on the health state 32211 (i.e., assuming that the combination of all problems would be acceptable). However, in calculating AHC_WORST_ we assumed that combinations would not be acceptable, so that severe problems with mobility (i.e. health state 31111) would be the lowest acceptable health state (Note: the most severe problem was determined by the utility score of each level in each domain, based on Lamers et al. [16, 17], not by the level itself).

In addition to the questions described above, respondents were presented with three specific health profiles (i.e. 21211, 22221 and 33312) and were asked to indicate from which age onwards they considered each of these health states to be acceptable. Based on Lamers et al. [16, 17], the utility values of these health profiles were 0.86, 0.69 and 0.20, respectively. We constructed a (partial) acceptable health curve, AHC_PROFILES_, based on the mean ages at which these QoL scores were considered acceptable, if considered acceptable at all. Respondents who indicated that complete health profiles were never acceptable were excluded from the means because these profiles were not considered to be acceptable at any age in the specified age range. Using t-tests, the mean ages at which the complete health profiles were considered acceptable were compared with an approximation of the ages at which they would be considered acceptable deduced from AHC_AGGREGATE_ and AHC_WORST_.

The area under the curve (AUC) was estimated for AHC_AGGREGATE_ and AHC_WORST_ as a proxy for the acceptable total amount of lifetime QALYs after the age of 40. The AUC was approximated using a Riemann integral, dividing the area into 6 rectangular areas at the age intervals [40; 45], [45; 55], [55; 65], [65; 75], [75; 85] and [85; 90]. The surface of each area was calculated as the width of an age interval multiplied by the mean acceptable quality of life score in that interval (for example, the width of 10 years in the interval 45 to 55 multiplied by the mean acceptable QoL at age 50). For the interval [40; 45] the mean QoL score at the age of 40 was used and for the interval [85; 90] the mean QoL at the age of 90 was used. Both the AHC_AGGREGATE_ and AHC_WORST_ curves started at the age of 40. The end points of AHC_AGGREGATE_ and AHC_WORST_ were defined by the reported acceptable age of death, meaning that AHC_AGGREGATE_ and AHC_WORST_ were cut off at the acceptable age of death when it was lower than 90 years and linearly extrapolated up to that age when it was higher than 90 years.

Linear regressions of the AUC estimation of AHC_AGGREGATE_ on respondents’ characteristics were conducted in order to identify characteristics that may be associated with respondents’ perception of acceptable health. Variables for age, gender, income level, employment and having children were included in a basic model, which was then extended with variables for own health, health related lifestyle and age of death of next of kin. Health variables were having (had) a severe disorder or chronic illness. Health related lifestyle variables were dummy variables for regular exercise and therefore meeting the Dutch norm for healthy physical activity [18], having a healthy diet (i.e. varied, not too much, not too fat, including fruits and vegetables for at least 6 days per week) and dummies for smoking and alcohol intake (on average, drinking alcohol at least 1 day per week). In addition, the complete model was also estimated separately with mean acceptable quality of life (i.e. mean of reported acceptable quality of life scores at the ages of 40 to 90) and acceptable length of life (i.e. the reported acceptable age of death) as dependent variables. Stata12 was used for all analyses.

## Results

Table 1 presents the study sample characteristics. Respondents were 43 years of age on average and 58 % was older than 40 years. The majority had a medium or high education level, and 53 % was employed or self-employed. Respondents reported to be in relatively good health with a quality of life of 0.85, but nonetheless about one third reported to have (had) either a chronic illness or a severe disorder, or both. For the majority of the respondents (53.7 %), the age of death of their next of kin fell in the range of 75 to 85 years. Our data was sampled on representativeness for the Dutch general public between the ages of 18 and 65 in terms of age, gender and education level. Comparison with population norms for the general public in the Netherlands (see Table 1) indicates that our respondents are slightly older and more highly educated, but overall can be considered reasonably representative for this population.

**Table 1:** Study sample characteristics (n = 1067)

Variable	Level	Sample statistic	General public in age category 18-65^a^
Age (mean, S.E.)		43.2 (0.42)	42.0
Gender (% female)		49.8	49.8
Education level (%)	Low (LO, LBO, MAO)	27.1	31.0
	Medium (MBO, HAO)	42.0	40.3
	High (HBO, WO)	30.9	27.8
Daily activity (%)	Employed/Self-employed	53.1	
	Unemployed/student/retired/other	46.9	
Household income, net per month (%)	Low (up to 1499 euro)	30.0	
	Medium (1500 – 2999 euro)	47.3	
	High (3000 euro or more)	22.7	
Marital status (%)	Married/Living together	64.3	
	Other	35.7	
Children (%)	No	39.8	
	Yes	60.2	
Health	EQ-5D-3L (mean, S.E.)	0.85 (0.01)	
	Chronic illness (%)	36.6	
	Severe disorder (%)	28.2	
Lifestyle (%)	Regular exercise	50.9	
	Healthy diet	47.6	
	Smoking	39.5	
	Alcohol intake	64.0	
Acceptable age of death (mean, S.E.)		83.3 (0.23)	
Age of death next of kin (%)	75 years or lower	19.4	
	From 75 to 85 years	53.7	
	Higher than 85 years	26.9	

^a^Mean age is based on statistics for the Dutch population between the ages of 18 and 65, gender and education level are based on statistics for the Dutch population between the ages of 15 and 65. All population statistics are based on the year 2010. Source: CBS statline, Centraal Bureau voor Statistiek, via http://statline.cbs.nl/Statweb/

Table 2 indicates that, in general, all levels of health problems were considered increasingly acceptable with progressing age. Only few respondents (≤13.8 %) considered mild problems in any domain of health to be acceptable below the age of 60. However, the majority of respondents considered mild problems in the domains mobility, usual activities and pain/discomfort acceptable from the age of 70 onwards, mild problems in the domain self-care acceptable from the age of 80 onwards and mild problems in the domain anxiety/depression acceptable from the age of 90 onwards. In all domains except anxiety/depression, fewer than 10 % of the respondents considered mild health problems to be never acceptable.

**Table 2:** Acceptability of less than perfect health states beyond a certain age in percentage of respondents (n = 1067)

Health domain	Severity of health problems	Dutch EQ-5D-3L utility tariff^a^	Acceptability of domain-specific health problems (cum %)						Never acceptable
			From age 40 onwards	From age 50 onwards	From age 60 onwards	From age 70 onwards	From age 80 onwards	From age 90 onwards	
Mobility	Mild problems	−0.036	2.8	5.8	22.1	65.9	92.0	96.8	3.2
	Confined to bed	−0.161	0.3	0.8	1.7	8.6	32.2	58.5	41.5
Self-care	Mild problems	−0.082	1.1	1.2	5.5	32.1	78.9	93.9	6.1
	Severe problems	−0.152	0.6	0.7	1.4	8.1	36.8	74.9	25.1
Usual activities	Mild problems	−0.032	1.8	3.1	13.7	52.0	86.7	95.4	4.6
	Severe problems	−0.057	0.8	1.3	3.4	13.2	44.2	75.5	24.6
Pain/discomfort	Moderate	−0.086	6.4	13.8	32.2	62.5	84.9	90.2	9.8
	Extreme	−0.329	1.9	2.4	6.1	17.7	44.5	63.3	36.7
Anxiety/depression	Moderate	−0.124	6.7	9.3	14.3	27.5	45.2	56.0	44.0
	Extreme	−0.325	3.1	3.8	5.9	11.3	24.0	36.8	63.2
Total	None		89.1	80.6	56.8	18.9	4.3	2.0	2.0
	At least one mild/moderate		10.9	19.4	43.2	81.1	95.7	98.0	45.8
	At least one severe/extreme		4.3	5.2	10.5	27.8	63.3	87.5	68.9

^a^In order to attain EQ-5D-3L utility scores for health states, the standard deduction for any deviation from health state ‘11111’ (−0.071) and the deduction for having severe problems on at least one domain (−0.234) should be taken into account in addition to the domain-specific scores presented

Only few respondents (≤6.1 %) considered severe health problems to be acceptable below the age of 70. However, the majority of respondents considered severe problems in all domains except anxiety/depression to be acceptable from the age of 90 onwards. In all domains except anxiety/depression, fewer than 42 % of the respondents considered severe health problems never to be acceptable.

Even fewer respondents (2.0 %) considered none of the health problems acceptable at any of the ages of 40 to 90 (as presented in the last column of Table 2). This indicates that their reference point for acceptable health was perfect health in each domain (health profile 11111), i.e. no health problems in any of the five domains, up to the age of 90. This 2 % is also the only group of respondents who reported the same level of health problems to be acceptable across all ages. The remaining 98 % of respondents considered some level of health problems to be acceptable at some age, and differentiated between age categories in the level of health problems they considered acceptable.

**Fig. 1:**
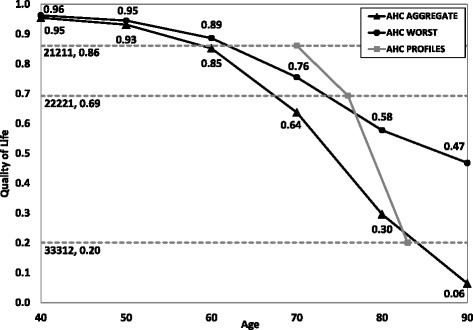
Acceptable health curves. Based on health problems on 5 domains (AHC_AGGREGATE_), health problems on 1 domain (AHC_WORST_) and complete health profiles (AHC_PROFILES_)

Figure 1 presents AHC_AGGREGATE_, AHC_WORST_ and AHC_PROFILES_. All curves are downward sloping, indicating that the acceptable quality of life diminishes with progressing age. AHC_AGGREGATE_ and AHC_WORST_ show a comparable decline up to the age of 60 years and start to diverge considerably from the age of 70 years onwards. At the age of 90, a QoL score of 0.06 is considered to be acceptable when health problems are aggregated on all 5 domains (i.e. AHC_AGGREGATE_), whereas a QoL score of 0.47 is considered to be acceptable according to the more conservative approach (i.e. AHC_WORST_). The areas under the curve were 31 QALYs for AHC_AGGREGATE_ and 35 QALYs for AHC_WORST_.

AHC_PROFILES_ presents the ages at which the three complete health states 21211, 22221, and 33312 (corresponding with QoL scores of 0.86, 0.69 and 0.20) were considered acceptable. Compared to this curve, AHC_AGGREGATE_ and AHC_WORST_ underestimated the ages at which the health states 21211 and 22221 were considered to be acceptable, but overestimated the age at which the health 33312 was considered to be acceptable. The mean age at which respondents reported health state 21211 to be acceptable was 70 years, which significantly differed from the mean ages of 59 and 62 years at which a QoL score of 0.86 appeared to be acceptable based on AHC_AGGREGATE_ and AHC_WORST_ (p < 0.001 for both 59 and 62 years; n = 1037). The mean age at which respondents reported health state 22221 to be acceptable was 76, which significantly differed from the mean ages of 67 and 74 years at which a QoL score of 0.69 appeared to be acceptable based on AHC_AGGREGATE_ and AHC_WORST_ (p < 0.001 for both 67 and 74 years; n = 1015). The mean age at which respondents reported health state 33312 to be acceptable was 83 years, which did not differ significantly from the mean age of 84 years at which a QoL score of 0.20 appeared to be acceptable on AHC_AGGREGATE_ (p = 0.100; n = 671). The QoL score of 0.20 was not on AHC_WORST_ because on this curve, the acceptable QoL did not fall below 0.47.

Table 3 presents the results of the linear regression analyses. The basic model contains the variables age and age squared, which were both significant, indicating that the relationship between age and the calculated area under the curve (AUC) of AHC_AGGREGATE_ was nonlinear. The joint effect of age and age squared was significant (Wald test, F = 13.19 p < 0.001) and negative between the ages of 18 and 65. On average, the area under the curve decreased with the age of the respondents, indicating that older respondents considered more health deterioration (in quality and/or length of life) acceptable at older age than younger respondents. The dummy variables gender and having children were not significant, but both income variables and the employment variable were. The effect of having a middle or low income as compared to a high income was negative. Hence, respondents with a low or middle income considered more health deterioration acceptable at older age than respondents with a high income. The effect of being employed was positive, indicating that employed or self-employed respondents considered less health deterioration acceptable at older age.

**Table 3:** Linear regressions of the area under the curve (AUC) of AHC on respondent characteristics (n = 1067)

	Basic model		Basic model & health		Basic model & health, lifestyle		Basic model & health, lifestyle, and life expectancy		Mean acceptable quality of life		Acceptable age of death	
	Coef.	S.E.	Coef.	S.E.	Coef.	S.E.	Coef.	S.E.	Coef.	S.E.	Coef.	S.E.
Age	-0.43^*^	0.17	-0.34^*^	0.17	-0.29	0.17	-0.25	0.17	-0.01	0.00	0.15	0.12
Age sq.	0.01^**^	0.00	0.01^**^	0.00	0.01^*^	0.00	0.00^*^	0.00	0.00	0.00	-0.00	0.00
Gender (male)	0.52	0.65	0.41	0.64	0.42	0.65	0.52	0.64	0.01	0.01	-0.54	0.46
Income low	-1.88^*^	0.91	-1.55	0.91	-1.08	0.91	-0.93	0.90	-0.02	0.02	0.22	0.65
Income medium	-1.98^*^	0.80	-1.89^*^	0.80	-1.63^*^	0.79	-1.42	0.78	-0.03	0.02	-0.65	0.56
Employed	2.14^**^	0.72	1.51^*^	0.73	1.42	0.73	1.33	0.71	0.03	0.02	-0.86	0.51
Children	1.02	0.74	0.73	0.74	0.97	0.73	1.08	0.72	0.01	0.02	0.56	0.52
Severe disorder			-2.14^**^	0.81	-2.03^*^	0.80	-1.78^*^	0.79	-0.02	0.02	-1.14^*^	0.57
Chronic illness			-1.61^*^	0.77	-1.71^*^	0.77	-1.59^*^	0.75	-0.03	0.02	-0.67	0.54
Regular exercise					0.61	0.64	0.46	0.63	-0.00	0.01	0.35	0.45
Healthy diet					2.68^***^	0.67	2.34^***^	0.66	0.06^***^	0.01	0.38	0.47
Smoking					-0.91	0.64	-1.00	0.63	-0.01	0.01	-0.92^*^	0.46
Alcohol intake					0.87	0.66	0.85	0.66	0.01	0.01	-0.13	0.47
Kin age low							-5.04^***^	0.91	-0.07^***^	0.02	-5.81^***^	0.66
Kin age medium							-3.80^***^	0.72	-0.07^***^	0.02	-2.65^***^	0.52
Constant	36.25^***^	3.17	35.42^***^	3.15	33.17^***^	3.22	35.70^***^	3.20	0.69^***^	0.07	82.78^***^	2.30
Adj R-squared		0.04		0.05		0.07		0.10		0.06		0.10

*** < .001; ** < .01; * < .05

The coefficients for the variables severe disorder and chronic illness in the second model were significant and negative in sign, indicating that respondents who reported to have (had) a severe disorder or chronic illness considered more health deterioration acceptable at older age. The interaction of severe disorder and chronic illness was not significant (p = 0.312, not shown). Compared to the basic model, the coefficient for low income was no longer significant in the second model and the coefficient for employment decreased in significance and magnitude. From the lifestyle variables added in the third model, the coefficient for having a healthy diet was positive and significant, indicating that respondents who reported to have a healthy diet considered less health deterioration acceptable at older age than respondents who did not meet this criterion. Compared to the second model, the significant effect of employment disappeared in the third model, while the joint effect of age and age squared was still significant (F = 9.22, p < 0.001). The coefficients for kin age in the fourth model were negative and significant. Hence, respondents whose next of kin die at higher ages considered less health deterioration acceptable at older age. The significant joint effect of age and age squared (F = 8.10, p < 0.001) turned positive at the age of 61, whereas it was negative for the entire age range of 18 to 65 in the basic, second and third model. Compared to the third model, having a middle income was no longer significant as a determinant of the AUC.

The last two columns of Table 3 present the models with mean acceptable quality and length of life as dependent variables. In the acceptable quality of life model, as compared to model 4, having a healthy diet and age of death of next of kin remained significant. In the acceptable length of life model, as compared to model 4, severe illness and age of death of next of kin remained significant. In addition, the coefficient of smoking was negative and significant, indicating that those who smoke considered a shorter length of life acceptable.

## Discussion

In this paper, we have empirically tested the notion of the acceptability of non-perfect health states, which may be relevant in the context of priority setting in health care. Our results indicate that some non-perfect health states are considered to be acceptable and that the acceptability of health problems in general increases with progressing age. At the ages of 40, 50, and 60 only few respondents considered any level of health problems to be acceptable, but the proportion that considered mild health problems acceptable increased considerably from the age of 70 years onwards. A majority of respondents considered mild problems in any health domain to be acceptable from the age of 70 onwards, except for self-care and anxiety/depression. Perhaps this age pattern is related to the common (albeit changing) pensioning age in the Netherlands of 65 years. Problems that prevent people from actively participating may be less often considered acceptable when people have yet to reach the pensioning age. Severe problems were considered acceptable by the majority of respondents from the age of 90 onwards, except in the domain anxiety/depression. As expected, at any given age severe health problems were less often considered acceptable than mild health problems. Moreover, the frequency of ‘never acceptable’ was considerably higher for severe problems than for mild health problems in all health domains. For example, 3 % of respondents considered mild problems with mobility to be never acceptable whereas 42 % considered being confined to bed to be never acceptable. Therefore, our results suggest that the distinction between unacceptable and acceptable health problems may be particularly relevant for milder health problems.

A remarkable feature of our results is the deviating pattern of responses in the domain anxiety/depression. The proportion of respondents that considered mild or severe health problems in this domain to be never acceptable was substantially higher than in the other four domains. This is in line with the earlier findings of Brouwer et al. [12] and societal preferences according to the Dutch EQ-5D-3L value set [17]. These findings suggest that people may be particularly averse to problems with anxiety/depression and that they are generally considered to be less acceptable than problems in other domains, regardless of age.

The regression analysis also yielded some noteworthy results. First, the negative effect of age on the area under the curve (AUC) of AHC_AGGREGATE_ indicates that older respondents considered more health deterioration (in quality and/or length of life) acceptable than younger respondents. Perhaps older respondents considered a lower quality of life more acceptable at older ages because they better understand how to cope with a certain extent of health deterioration over time. They may also consider the health and life expectancy of their peers or next of kin as a reference, and this reference point may change as these reference groups get older (or die). Having a chronic illness lowered the AUC by 1.6 QALYs, while having (had) a severe disorder lowered it by 2.1 QALYs. This indicates that these subgroups found a considerably lower amount of lifetime QALYs after the age of 40 years acceptable. Taken together, these findings suggest that experience with health problems – through ageing, chronic illness or a severe disorder – and better understanding of their impact on normal functioning at older age possibly influenced respondents’ perception of what is acceptable. Accordingly, they considered less than perfect health states acceptable more often than other respondents did.

Second, respondents with a healthy diet reported a significantly higher AUC (2.7 and 2.3 QALYs in the third and fourth model respectively) than those with an unhealthy diet. In the separate acceptable quality and length of life models, we found a significant positive effect of having a healthy diet on acceptable quality of life (0.06), but not on acceptable length of life. The higher AUC, therefore, seems primarily induced by acceptable QoL considerations. For smoking, the opposite was found: acceptable length of life was almost 1 year shorter for smokers than for non-smokers, while no effect of smoking was found on acceptable QoL or AUC. These findings indicate that people may adapt their perception of what is acceptable based on their lifestyle choices at the level of length or quality of life, but not necessarily the combination of the two, based on their understanding that some unhealthy behaviours are associated with (chronic) illness while others are associated with (earlier) death.

Third, the AUC was 5.0 and 3.8 QALYs lower for respondents whose next of kin died before the age of 75 or between the ages of 75 and 85, respectively. This effect was found for both quality and length of life, despite the fact that age of death of next of kin is a measure of length rather than quality of life. This may indicate some correlation between length and quality of life (e.g. that the quality of life of next of kin who die younger was lower).

### Limitations

Some limitations of our study need to be addressed. First, caution is warranted in generalising our results. Although our sample was representative for the population of the Netherlands between the ages of 18 and 65 in terms of age, gender and education level, none of our respondents was older than 65. This means that we have excluded the elderly population entirely from this study, while these are the people who have most experience with ageing and coping with related health problems. Their perception of what is acceptable may be substantially different from that of our (i.e. younger) respondents. Therefore, an interesting avenue for future research may be to focus on investigating the acceptability of less than perfect health states in an elderly population. In addition, respondents were contacted by a sampling agency after voluntarily singing up to participate in scientific research, which may have resulted in selection bias.

Second, respondents assessed the acceptability of health problems in each domain separately, as already mentioned in the methods section. Evaluating a health problem in isolation does not immediately reveal how people would evaluate health profiles in which combinations of problems occur. Hence, we used two approaches (one additive and one restrictive) to calculate the AHCs. Since we also investigated the acceptability of three health profiles, we can compare the two curves to this method. The results showed that for the two milder health profiles 21211 and 22221, both AHC_AGGREGATE_ and AHC_WORST_ were below AHC_PROFILES_. For the worst health profile 33312, AHC_AGGREGATE_ and AHC_PROFILES_ were almost equal. AHC_WORST_ was constructed as a more conservative curve than AHC_AGGREGATE_, and for health states 21211 and 22221, AHC_WORST_ was closer to AHC_PROFILES_ than AHC_AGGREGATE_ was, but for 33312 AHC_AGGREGATE_ was closer to AHC_PROFILES_ than AHC_WORST_ was. The exact location of an acceptable health curve using complete health states therefore remains unknown and deserves more research. However, based on the results presented here we do have some understanding of its shape. The finding that the ages at which the health states 21211, 22221 and 33312 were considered acceptable were consecutively higher is in line with the downward-sloping AHC_AGGREGATE_ and AHC_WORST_ with age. A recommendation for future research may be to estimate the acceptable health curve with a larger set of complete health profiles, rather than with health problems in isolation.

Third, the concept ‘acceptable health’ was not further defined than the health state that may be considered ‘normal’ for someone at a certain age. Without further explanation of the concept of acceptable or normal health, respondents may have interpreted acceptability differently. For example, ‘acceptable’ or ‘normal’ may have been interpreted as the health needed to live a decent life, the health needed to live a minimally tolerable life, some average indication of actual health at older ages or the health needed to maintain one’s standard or aspiration of life. In addition, the questions did not specify whether respondents should assess what they consider acceptable for themselves or for a random other person in society. However, the effect of respondents’ lifestyle choices on their perceptions of acceptability that were found in the regression analyses suggests that respondents indicated what they considered to be acceptable for themselves. Future studies could shed more light on the relevance of these aspects, for instance, by adding follow-up questions asking respondents for their interpretation, by choosing for more specific formulations of the questions, or by experimenting with different formulations.

Finally, the results of this study are descriptive and not normative in nature. Hence, we may conclude that acceptability *can,* in theory, be used to distinguish between health problems in allocating resources, but it does not allow us to conclude that acceptability *should* be used in priority setting in health care. The normative justification of using acceptable levels of health in this context requires further attention. Future research may focus on this normative justification and the ethical implications of using acceptability as a means of differentiating treatments. Special attention may be put on the ageist implications of (this specific operationalisation of) acceptable health. That is, acceptability-based rationing implies an age-bias towards the young, which may be seen as unfair. It should be noted however, that there is also a considerable body of literature on ageist preferences and support for the ‘fair innings’ argument [5–7].

### Implications

If acceptable health is applied as a reference point in priority setting in health care, the societal value attached to health gains may differ based on their position relative to this reference point. Treatments directed at unacceptable health problems (or patients in unacceptable health states) may be assigned higher priority than those directed at acceptable health problems (or patients in acceptable health states). The value assigned to health gains below and above the reference level of acceptable health may then lead to differential weighting of a health gain of the same size between people of the same age (because levels of health before treatment differ) as well as between people of different ages (because the reference levels of acceptable health differ). An interesting avenue for future research is to investigate how health gains above the reference level of acceptable health are traded off against those below this reference level. In addition, it may be interesting to investigate whether, and if so, to what extent, the distance to the reference level of acceptable health within groups of acceptable or unacceptable health problems influences health state valuations. In this context, it is noteworthy that only 2 % of our sample considered none of the health problems acceptable at any of the ages of 40 to 90 and therefore had a reference point of perfect health for all age groups, in line with the common QALY framework. Shifting the reference point in priority setting from perfect health to acceptable health therefore is likely to have implications for the value attached to health gains according to a very large majority of the population (i.e. 98 %).

## Conclusion

Based on the findings of this study we may conclude that there seem to be age-dependent levels of non-perfect health that are considered acceptable by the general public. Consequently, as people get older, an increasing proportion of their health problems may be expected to fall in a range that is considered to be acceptable in relation to their normal functioning, and may thus receive lower weight in priority setting from the perspective of the general public. Future research may be aimed at investigating whether assigning different weights to treatments above and below this threshold is indeed in line with social preferences. After all, given persistent scarcity in health care resources, rationing according to social preferences is essential for achieving health gains in a way that is generally considered fair.

## Appendix

### Survey instrument (translated from Dutch)

General introduction

In what follows, you receive questions regarding health at different ages. Could you please indicate which health state you consider to be acceptable at different ages. For example, consider an 80-year old person and a 20-year old person who are both unable to walk more than 1 km per day. You might consider this acceptable for an 80-year old person, because it is normal for elderly not being able to walk very far. You might consider this less acceptable for a 20-year old person, because a 20-year old person should generally be able to walk 1 km per day.

The following question was posed for all five EQ-5D-3L domains:

**Table:**

Could you please indicate from which age onwards you consider problems with mobility acceptable?							
	*From 40 onwards*	*From 50 onwards*	*From 60 onwards*	*From 70 onwards*	*From 80 onwards*	*From 90 onwards*	*Never*
Some problems in walking about	□	□	□	□	□	□	□
Confined to bed	□	□	□	□	□	□	□

## Abbreviations

QALY: Quality-adjusted life year
ICUR: Incremental cost-utility ratio
QoL: Quality of life
AHC: Acceptable health curve
AUC: Area under the curve

## References

[1] Drummond MF, Sculpher MJ, Torrance GW, O’Brien B, Stoddart GL (2005). Methods for the economic evaluation of health care programmes: 3rd ed. Oxford medical Publications 1997.

[2] Weinstein MC, Torrance G, McGuire A (2009). QALYs: the basics. Value in health.

[3] Bobinac A, van Exel NJA, Rutten FFH, Brouwer WBF (2013). Valuing QALY gains by applying a societal perspective. Health Econ.

[4] Claxton K, Paulden M, Gravelle H, Brouwer W, Culyer AJ (2011). Discounting and decision making in the economic evaluation of health-care technologies. Health Econ.

[5] Dolan P, Shaw R, Tsuchiya A, Williams A (2005). QALY maximisation and people's preferences: a methodological review of the literature. Health Econ.

[6] Schwappach DL (2002). Resource allocation, social values and the QALY: a review of the debate and empirical evidence. Health Expect.

[7] Van de Wetering E, Stolk E, Van Exel N, Brouwer W (2013). Balancing equity and efficiency in the Dutch basic benefits package using the principle of proportional shortfall. Eur J Health Econ.

[8] Williams A (1997). Intergenerational equity: an exploration of the'fair innings' argument. Health Econ.

[9] Nord E, Pinto JL, Richardson J, Menzel P, Ubel P (1999). Incorporating societal concerns for fairness in numerical valuations of health programmes. Health Econ.

[10] Nord E (2005). Concerns for the worse off: fair innings versus severity. Soc Sci Med.

[11] Nord E, Johansen R (2014). Concerns for severity in priority setting in health care: A review of trade-off data in preference studies and implications for societal willingness to pay for a QALY. Health Policy.

[12] Brouwer WBF, Van Exel N, Stolk EA (2005). Acceptability of less than perfect health states. Soc Sci Med.

[13] Stolk EA, Brouwer WB, Busschbach JJ (2002). Rationalising rationing: economic and other considerations in the debate about funding of Viagra. Health Policy.

[14] Tversky A, Kahneman D (1981). The framing of decisions and the psychology of choice. Science.

[15] EuroQol Group (1990). EuroQol-a new facility for the measurement of health-related quality of life. Health policy.

[16] Lamers LM, McDonnell J, Stalmeier PFM, Krabbe PFM, Busschbach JJV (2006). The Dutch tariff: results and arguments for an effective design for national EQ‐5D valuation studies. Health Econ.

[17] Lamers L, Stalmeier P, McDonnell J, Krabbe P, Busschbach J (2005). Kwaliteit van leven meten in economische evaluaties: het Nederlands EQ-5D-tarief. Ned Tijdschr Geneeskd.

[18] Kemper H, Ooijendijk WMT, Stiggelbout M (2000). Consensus over de Nederlandse norm voor gezond bewegen. Tijdschrift voor Gezondheidswetenschappen.

